# Fundamental principles of an effective diabetic retinopathy screening program

**DOI:** 10.1007/s00592-020-01506-8

**Published:** 2020-03-28

**Authors:** Paolo Lanzetta, Valentina Sarao, Peter H. Scanlon, Jane Barratt, Massimo Porta, Francesco Bandello, Anat Loewenstein, the Vision Academy, Bora Eldem, Alex Hunyor, Antonia Joussen, Adrian Koh, Jean-François Korobelnik, Paolo Lanzetta, Anat Loewenstein, Monica Lövestam-Adrian, Rafael Navarro, Annabelle A. Okada, Ian Pearce, Francisco J. Rodríguez, Giovanni Staurenghi, Sebastian Wolf, David T. Wong

**Affiliations:** 1 Department of Medicine – Ophthalmology, University of Udine, Piazzale S. Maria della Misericordia, 33100 Udine, Italy; 2 Istituto Europeo di Microchirurgia Oculare (IEMO), Udine, Italy; 3 Gloucestershire Hospitals NHS Foundation Trust, Gloucester, UK; 4 International Federation on Ageing, Toronto, Canada; 5 Department of Medical Sciences, University of Turin, Turin, Italy; 6 San Raffaele Scientific Institute, Milan, Italy; 7 Department of Ophthalmology Tel Aviv Medical Center, and Sackler Faculty of Medicine, Tel Aviv University, Tel Aviv, Israel

**Keywords:** Diabetic retinopathy screening, Telemedicine, Evidence-based recommendations

## Abstract

**Background:**

Diabetic retinopathy (DR) is the leading cause of blindness among working-age adults worldwide. Early detection and treatment are necessary to forestall vision loss from DR.

**Methods:**

A working group of ophthalmic and diabetes experts was established to develop a consensus on the key principles of an effective DR screening program. Recommendations are based on analysis of a structured literature review.

**Results:**

The recommendations for implementing an effective DR screening program are: (1) Examination methods must be suitable for the screening region, and DR classification/grading systems must be systematic and uniformly applied. Two-field retinal imaging is sufficient for DR screening and is preferable to seven-field imaging, and referable DR should be well defined and reliably identifiable by qualified screening staff; (2) in many countries/regions, screening can and should take place outside the ophthalmology clinic; (3) screening staff should be accredited and show evidence of ongoing training; (4) screening programs should adhere to relevant national quality assurance standards; (5) studies that use uniform definitions of risk to determine optimum risk-based screening intervals are required; (6) technology infrastructure should be in place to ensure that high-quality images can be stored securely to protect patient information; (7) although screening for diabetic macular edema (DME) in conjunction with DR evaluations may have merit, there is currently insufficient evidence to support implementation of programs solely for DME screening.

**Conclusion:**

Use of these recommendations may yield more effective DR screening programs that reduce the risk of vision loss worldwide.

## Introduction

Diabetic retinopathy (DR) is a well-known complication of diabetes and the main cause of blindness among working-age adults [1]. Timely detection and treatment of DR can prevent blindness, but many people with this condition are not diagnosed early enough to be treated effectively. People with sight-threatening DR (STDR) are often asymptomatic during the period in which the condition is treatable by photocoagulation or intravitreal therapy [2]. Therefore, community-wide education and implementation of effective programs for DR screening are needed.

Numerous screening programs have been established throughout the world; these usually involve assessment and grading of the eyes of patients with diabetes, and referral of those with STDR to an ophthalmologist. The principles and considerations for screening programs, proposed by Wilson and Jungner on behalf of the World Health Organization in 1968 [3], have been adopted widely in practice, including in the English National Health Service (NHS) Diabetic Eye Screening Programme [4]. Since the implementation of this program in 2003, diabetic retinopathy/maculopathy is no longer the leading cause of blindness in the working population in England [4]. The International Council of Ophthalmology published the 2017 Guidelines for Diabetic Eye Care, which includes a guide to DR screening as well as follow-up and management based on different resource settings [5]. A successfully implemented screening program should benefit patients by increasing awareness of the importance of regular monitoring and providing a prompt referral to an ophthalmologist for screen-positive DR, to ultimately reduce the risk of sight loss and preserve patient function and quality of life [6–8].

Although established screening programs have reduced the risk of sight loss among people with diabetes worldwide, consensus among experts on effective screening in DR is lacking. Therefore, a working group of ophthalmic and diabetes experts was established and convened through the Vision Academy, a Bayer educational initiative, to propose evidence-based recommendations for screening for DR. This review will present these recommendations, assess unmet needs and identify areas for further investigation.

## Methods

### Searches

A structured literature search with predetermined search terms and inclusion/exclusion criteria was undertaken to identify studies worldwide that address the effectiveness of DR screening programs and screening tools. An initial search of MEDLINE/PubMed was performed on March 8, 2018, using the following search terms: “diabetic retinopathy” OR “diabetic macular edema” OR DME OR “diabetic macular oedema” OR DMO AND “screening program” OR screening OR “teleretinal screening” OR telemedicine OR teleophthalmology.

After screening study titles for relevance, abstracts were read, and studies that involved the following were included: (1) patients with diabetes, (2) a sample size of > 200 patients and (3) quality or cost-effectiveness of DR screening programs or telemedicine systems. Only studies published in the preceding 10 years (to March 8, 2018) were included, and those with the greatest scientific impact (average of ≥ 3 citations per year) were prioritized. No study exclusions were made on the basis of gender, age, disease severity, presence of comorbidities, socioeconomic status or geographic region.

After screening titles and abstracts, the full text from 231 articles were examined in detail and 89 articles of interest were identified for the review.

## Results

### Standard imaging techniques

As the pathology of DR includes characteristic microvascular lesions detectable on fundus examination, obtaining ophthalmic images is the primary method of screening for DR. The most typical imaging method used is retinal fundus photography (FP), but dilated slit-lamp biomicroscopy may be another option. Results can be assessed at the point of care or in a telemedicine setting. Imaging is performed by trained personnel (e.g., technicians, nurses, family physicians, optometrists or endocrinologists), and results can be evaluated manually by certified graders/readers (e.g., ophthalmologists, retina specialists or trained technicians) or automatically by image analysis algorithms.

Seven-field stereoscopic FP was recognized as the reference standard for assessing effectiveness of DR screening programs in the Early Treatment Diabetic Retinopathy Study (ETDRS) [4]. However, in a review of 45 studies, researchers noted that the most commonly used imaging method in DR screening was two-field fundus imaging [9].

Both mydriatic and non-mydriatic approaches are common in DR screening programs. Induction of mydriasis may improve specificity of DR detection, but involves longer examination time and greater patient discomfort. In a study of community-based screening for STDR, Baeza et al. (2009) determined that imaging one non-stereoscopic field with a non-mydriatic camera (NMC) was comparable in sensitivity and specificity to seven-field standard stereoscopic imaging, as long as mydriasis was performed. If NMC screening without mydriasis was performed, the percentage of patients referred to ophthalmologists increased from 5 to 15% because of ungradable photographs [10]. In a meta-analysis including data published up to June 2009, variations in mydriatic status did not significantly affect sensitivity or specificity of DR detection [11]. However, subsequent meta-analyses do not support this. The authors in India concluded that non-mydriatic digital imaging had low sensitivity and resulted in a high rate of ungradable images, particularly in a dark iris population [12]. Similar results were obtained in a pilot telescreening study in Italy that involved 22,466 patients [13]. In a meta-analysis of digital imaging-based telemedicine for DR screening, the researchers found that diagnostic accuracy was higher when images were obtained when mydriasis was performed versus when it was not, especially when a wide angle was used [14].

### Alternative imaging techniques

Non-mydriatic ultra-wide-field (UWF) imaging and optical coherence tomography (OCT) may be applicable in future DR screening efforts; work is ongoing to establish the validity and cost-effectiveness of these tools at the point of care and in a telemedicine setting [15–19].

**Fig. 1:**
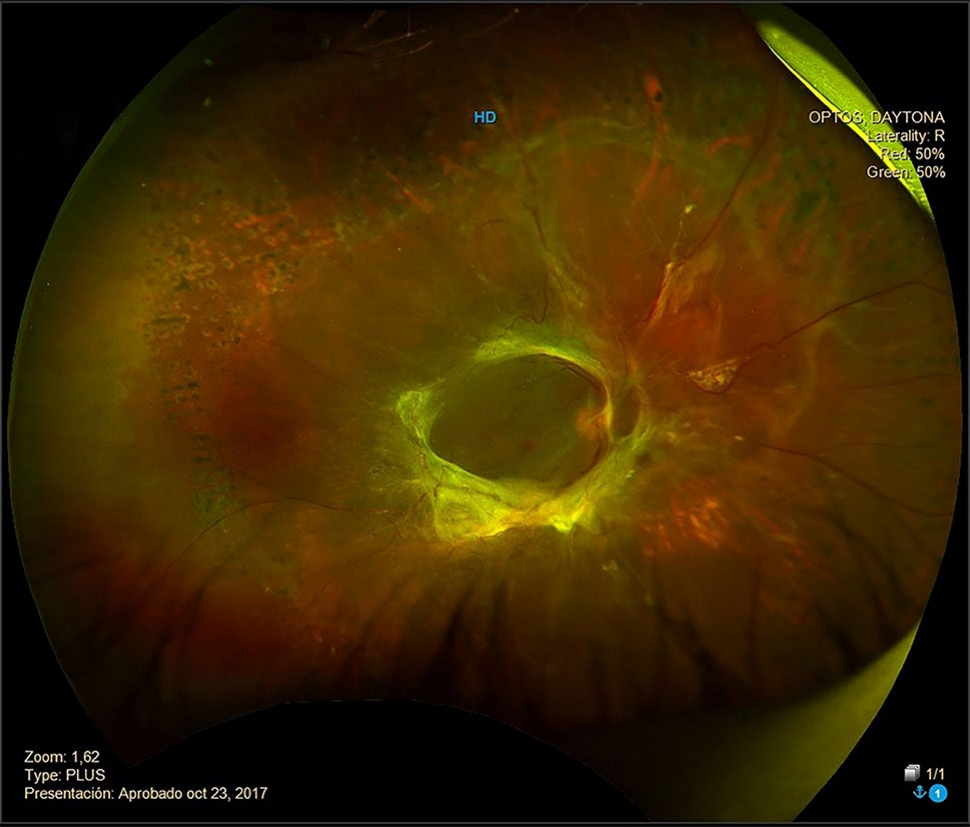
Example of a UWF fundus image showing significant fibrosis due to proliferative diabetic retinopathy

UWF applies the principles of scanning laser ophthalmoscopy (SLO) with an ellipsoid mirror to capture a 200° image versus the 45°–50° image obtained with standard FP. This permits imaging of the peripheral retina and observation of DR lesions that might otherwise be missed [16]. An example of an image obtained using UWF FP is shown in Fig. 1. A telemedicine program in which trained non-physicians performed UWF SLO imaging and immediate grading of minimal DR was found to have good sensitivity and specificity [18]. Compared with the reading center evaluation, real-time image evaluation had sensitivity of 0.95 (95% confidence interval [CI] 0.94–0.97) and specificity of 0.84 (95% CI 0.82–0.85) for minimal DR, and for referable DR (RDR) had sensitivity of 0.99 (95% CI 0.97–1.00) and specificity of 0.76 (95% CI 0.75–0.78) [18]. In addition, there were relatively few ungradable images (ungradable rate of 5.3% per eye) [18].

In large-scale DR teleophthalmology programs, results from non-mydriatic UWF SLO imaging were found to be superior to results from non-mydriatic multifield FP [17]. Specifically, UWF imaging reduced the number of ungradable eyes by 81%, increased DR detection twofold and enabled detection of predominantly peripheral lesions (which are suggestive of severe DR) in almost 10% of patients [17]. In a study that compared UWF with clinical examination alone, UWF imaging allowed for earlier diagnosis of more patients with higher-grade DR [20].

In a private multispecialty hospital in the United Arab Emirates, imaging with a UWF fundus camera by nursing personnel in the endocrinology department was an effective form of telemedicine for DR screening [19]. This method ameliorated the burden of screening for DR in the retina clinic and improved the rate of early detection of DR [19]. Although an early UWF SLO study required more time for image analysis, yielded a higher rate of ungradable eyes and failed to detect some foveal microaneurysms (MA) compared to a digital camera [21], a more recent study demonstrated that the peripheral lesions detected through UWF imaging showed that the level of DR was more severe in 10% of patients than shown by non-mydriatic multifield fundus imaging [17].

The value of OCT in conjunction with FP for DR screening is currently unclear. Unlike two-dimensional FP, OCT provides volume and thickness data with three-dimensional visualization of pathological changes related to DME and can be used to confirm or rule out DME. In cases where DME is confirmed, OCT provides objective quantitative data to help guide treatment decisions and management of DME. Disadvantages of OCT include equipment costs and the need for a highly skilled operator to interpret images and determine imaging artifacts [15]. OCT is not advocated in primary screening because the price of the OCT equipment makes it highly unlikely that it will be cost-effective, given that in most screening studies at least 65% of the population with diabetes do not have DR. Advocates for the use of OCT currently recommend that it is only used to confirm DME when there is evidence of diabetic maculopathy from standard digital color photographs and two-dimensional markers. This approach has been shown to be cost-effective [22, 23].

Smartphone-based imaging tools may be useful for DR screening programs in which cost and availability of trained eye care personnel are barriers to implementation [24, 25]. However, no handheld device has been found to have comparable sensitivity and specificity to seven-field stereoscopic photography in detection of STDR. Rajalakshmi et al. [26] compared the “fundus on phone” (FOP) smartphone-based retinal camera with seven-field digital retinal photography and noted that the modalities yielded the same results for 92.7% of patients (kappa, 0.90). These authors suggested that the FOP camera was effective for screening and diagnosis of DR and STDR [26]. However, they noted that all patients underwent mydriasis in this study and that the image quality of the reference standard was superior to that of the FOP system. In this study, the smartphone was fixed and the patient’s head was secured by using a slit-lamp chin rest. This simple variation may significantly improve results from the use of smartphone technology because it reduces any image blurring caused by movement of the operator and/or the patient. More research is needed in terms of multisite trialing, measurement of the impact of smartphone-based DR assessment on clinical workflow and determination of the effects of this technology on health outcomes of screened patients [24].

### Functional assessments

At present, visual acuity is the most widely used functional measure in routine DR screening. Loss of visual acuity may occur at different stages of DR, but visual acuity alone is not a reliable measure for predicting DR. For example, in a study of 1549 patients with diabetes, the sensitivity and specificity of using subnormal vision to screen for STDR were 33.4% and 85.9%, respectively [27], which are well below recommended thresholds of 80% and 95%, respectively [28]. Measuring visual acuity alone may therefore not be sensitive enough to detect early DR so should only be used in conjunction with imaging methods during screening. However, as stereoscopic imaging facilities may not be available in all centers, in some circumstances it could be necessary to rely only on visual acuity measures for detecting potential DR. Other measures (including contrast sensitivity, dark adaptation and electrophysiologic parameters) can be helpful endpoints in translational research [29]. However, more research is warranted to determine whether measures other than visual acuity have any utility in DR screening [29].

### Grading disease severity

Different scales are used for grading the severity of DR. The ETDRS scale has 11 grades, from no disease to advanced proliferative DR (PDR) [30]. Although this scale is useful for research purposes, it is more complex than what is needed for DR screening programs, where the goal is to identify patients for referral and treatment. Accordingly, grading systems that have fewer grades and therefore simplify classification have been developed for DR screening. Typically, these scales acknowledge the four stages of DR: mild non-proliferative DR (NPDR), moderate NPDR, severe NPDR and PDR [31]. Examples and definitions of the different grades are shown in Table 1 [5, 32, 33].

**Table 1:** Examples of grading scales for DR

ETDRS grading (level)	ICO grading	AAO grading	RCOphth grading
No disease (10)	No abnormalities	No abnormalities	No disease
MA only (20)	Mild NPDR MA only	Mild NPDR MA only	Low risk
Mild NPDR (35)	Moderate NPDR MA + other signs, excluding those indicating severe NPDR	Moderate NPDR MA + other signs, but none defining severe NPDR	–
Moderate NPDR (43)	–	–	High risk
Moderately severe NPDR (47)	–	–	–
Severe NPDR (53A–D)	Severe NPDR >20 intraretinal hemorrhages in all quadrants Venous beading in two or more quadrants IRMA in one or more quadrant	Severe NPDR Intraretinal hemorrhages in all quadrants Venous beading in two or more quadrants IRMA in one or more quadrant	–
Very severe NPDR (53E)	–	–	–
Mild PDR (61)	PDR Neovascularization and/or vitreous/preretinal hemorrhage	PDR Neovascularization and/or vitreous/preretinal hemorrhage	PDR
Moderate PDR (65)	–	–	–
High-risk PDR (71, 75)	–	–	–
Advanced PDR (81, 85)	–	–	–

*AAO* American Academy of Ophthalmology, *DR* diabetic retinopathy, *ETDRS* Early Treatment Diabetic Retinopathy Study, *ICO* International Council of Ophthalmology, *IRMA* intraretinal microvascular abnormalities, *MA* microaneurysms, *NPDR* non-proliferative diabetic retinopathy, *PDR* proliferative diabetic retinopathy, *RCOphth* Royal College of Ophthalmologists

In a meta-analysis, Bragge et al. (2011) noted inconsistencies among DR classification schemes used as a basis for referral [11]. According to the International Clinical DR and DME Disease Severity Scales, developed by the Global Diabetic Retinopathy Project Task Force on behalf of the American Academy of Ophthalmology (AAO) in 2002, any level of retinopathy more severe than mild retinopathy may warrant examination by an ophthalmologist [32]. This means that, according to the AAO definition of mild NPDR, a referral is needed for anything more than “microaneurysms only” (ETDRS level > 20) [32]. However, in the Royal College of Ophthalmologists (RCOphth) grading system, “low risk” equates to the AAO definition of mild NPDR, but the RCOphth referral for “high risk” is recommended at a more advanced stage (ETDRS level of 43) than the equivalent AAO referral (Table 1) [5, 32, 33]. These observations underscore that a universal feature-based classification/grading system for discerning DR is lacking.

### Telemedicine-based screening

Telemedicine is essentially the ability to locally capture digital data (including images) and send the files to a centralized location for evaluation. Telemedicine systems based on digital fundus imaging offer a feasible and efficient means to screen patients who are not being reached by screening efforts in the specialist’s office [2]. A review of the literature confirmed that telemedicine has the potential to modify patient behaviors, which would contribute to diabetes control and prevention [34]. Telemedicine screening may take place at a diabetes clinic; in the primary care offices of a physician, optometrist or pharmacist; at university hospitals; or in mobile units.

In a randomized controlled trial, Mansberger et al. [35] compared the effectiveness of DR telescreening with an NMC versus traditional surveillance by an eye care provider. The authors determined that patients in the telemedicine group were more likely to undergo DR screening in the first year of enrollment [35]. Two years into the study, telemedicine-based screening was offered to all patients [36]. The authors’ 5-year findings indicate that telemedicine is an effective means of initial DR screening and monitoring for DR progression [36]. In a hypothetical cohort of unscreened patients with type 2 diabetes, a telemedicine-based DR screening model with trained technicians had lower costs than a physician-based model and produced similar quality-adjusted life years (QALYs) from a societal perspective [37].

DR screening with a mobile NMC can be beneficial for reaching patients who otherwise would not be included in a recommended screening program [38]. Cuadros and Bresnick [39] noted that patients tend not to seek annual DR screening and suggested incorporating telemedicine screening into primary care practices; these authors applied EyePACS, a Web-based means of retinopathy grading. Results from an Australian study showed that general practice-based DR screening is effective, enabling improved recording of screening outcomes and facilitating better follow-up of patients with RDR [40]. In a review, Das et al. [41] determined that DR is highly suitable for telemedicine because it saves time and minimizes lost income for the patient. DR telescreening can also provide cost savings for health systems depending on the population being screened (see the “Cost-effectiveness of screening” section); for example, a review highlighted that remote and underserved areas benefited most, with one rural teleophthalmology program resulting in savings of $150 per patient over 7 years [42].

Based on a literature review, Surendran and Raman [43] concluded that telescreening for DR is cost-effective, accurate and reliable, and that digital imaging systems are safe and effective alternatives to dilated indirect ophthalmoscopy coupled with biomicroscopy or stereoscopic FP. Different countries have different health service systems in place, and financial support for screening programs can be difficult to secure. Charitable sources, such as the Queen Elizabeth Diamond Jubilee Trust, provide grants to fund DR screening programs in less developed nations [44].

Pareja-Ríos et al. [45] reviewed 8 years of experience in teleophthalmology screening (Retisalud program; Canary Islands) and found that the number of patients screened per year increased steadily; the waiting times for image assessment decreased, and the ability of family doctors to correctly interpret retinographies improved progressively. In addition, the rate of ungradable retinographies decreased—partly owing to incorporation of mydriasis—and the percentage of images graded as normal increased [45]. Moreover, cases of severe NPDR and PDR constituted 14% initially and 3% at the end of the study. These findings underscore the fact that the full benefits of a DR screening program can take several years to develop [45].

### Staff accreditation and training

An effective DR screening program should include an accreditation system and require staff to demonstrate evidence of ongoing training. In the English NHS Diabetic Eye Screening Programme, all screeners and graders must show evidence of continuing professional development and take monthly external quality assurance tests. An international version of the accreditation and tests are also available for those working outside the UK [4].

Trained primary care physicians have been found to grade retinal photographs with acceptable accuracy when compared with ophthalmologists. In a study by Farley et al. (2008), the trained clinicians failed to refer 35 (10.2%) of the 344 patients that the ophthalmologist believed needed referral, which was concluded to be reasonable [46]. Romero et al. (2010) found grading concordance in terms of DR, DME and macular lesions between family physicians and reference ophthalmologists; the authors concluded that involving family physicians in DR screening can be effective [47]. In a retrospective, cross-sectional study in Singapore, trained non-physician graders proved to be a satisfactory and cost-effective alternative to family physicians for detecting DR [48].

### IT infrastructure

DR screening programs should be supported by a strong IT infrastructure that safeguards patient data and effectively balances image quality and file size. A typical DR screening program will require approximately 80,000 images to be stored per year. Although file size used to be a significant constraint [49], more recently file storage technology has improved and all files can be stored on a single server. The English NHS Diabetic Eye Screening Programme recommends that images captured are compressed to 1 to 2 MB [4].

### Automated disease detection

The diagnostic performance of artificial intelligence (AI)-based software for DR detection has been evaluated in several studies (Table 2). Deep learning (DL) is an AI-based application whereby convolutional neural networks (CNNs) train themselves to interpret images by iterative analysis and comparison of the output with a reference standard (i.e., diagnosis by human graders). The process is repeated by the CNN until the diagnostic output agrees with the reference standard. DL algorithms have been developed for the automated detection of DR lesions from color fundus photographs—these systems are being implemented in screening programs and have the potential to reduce screening costs and increase service efficiency in healthcare economies in the developed world and to aid delivery of DR screening in developing and remote settings [16, 50–54]. Retinal fundus imaging with an NMC and automated grading take only 10 minutes, can be performed at the point of care and may obviate a separate visit to an ophthalmologist [55]. Automated analysis of OCT images through use of DL is being explored in a collaborative project between Moorfields Eye Hospital and Google DeepMind [56], and in other retinal centers of excellence [57].

**Table 2:** An overview of advances in automated DR screening from 2008 to 2018

References	Type of technology	Sample size	Outcome measures			Author comments
			Sensitivity	Specificity	Study-specific outcome measures	
Abràmoff et al. [65]	Preliminary study to determine how a combination of algorithms for automated detection of DR compares with the clinical evaluation of a retina specialist	5692 patients	84%	64%	AUROC^a^: 0.84 Number needed to miss: 80	–
Abràmoff et al. [55]	IDP	874 patients	96.8%	59.4%	6/874 false negatives NPV: 98.5% PPV: 39.8% AUROC: 0.937	–
Solanki et al. [66]	EyeArt image analysis customized for DR screening and engineered for large-scale cloud deployment	874 patients (1748 eyes)	93.8%	72.2%	AUROC^a^: 0.941 False negatives: 22 (moderate NPDR; did not meet treatment criteria) No cases of macular edema were missed	–
Abràmoff et al. [67]	IDP with enhanced deep learning	874 patients	96.8%	87.0%	6/874 false negatives NPV: 99.0% PPV: 67.4%	This hybrid screening algorithm (IDx-DR) was the first AI device to obtain FDA approval for DR screening (in April 2018)
Gulshan et al. [68]	DL-trained algorithm, validated using two data sets (EyePACS-1 and Messidor-2)	128,175 images from 5871 patients (EyePACS-1: 4997; Messidor-2: 874)	EyePACS-1: 97.5% Messidor-2: 96.1%	EyePACS-1: 93.4% Messidor-2: 93.9%	**AUROC**^**a**^ EyePACS-1: 0.991 Messidor-2: 0.990	–
Gargeya and Leng [69]	DL algorithm	75,137 images	94%; Additional testing with Messidor-2 data set: 93%	98%; Messidor-2: 87%	AUROC^a^: 0.97 Messidor-2: 0.94	–
Ting et al. [70]	DL system	14,880 patients; 71,896 images from primary validation data set and 10 multiethnic cohorts with diabetes	90.5%	91.6%	AUROC^a^: 0.889–0.983	–
Tufail et al. [52]	EyeArt, Retmarker, iGradingM	20,258 patients	EyeArt: 94.7% Retmarker: 73.0% iGradingM: 100%^b^	EyeArt: 20% Retmarker: 52.3% iGradingM: –^b^	EyeArt false positive: 80.1% Retmarker false positive: 47.7% iGradingM false positive: 100%^b^	Retmarker and EyeArt systems show acceptable sensitivity for RDR when compared with human graders and have sufficient specificity to make them cost-effective alternatives to manual grading alone
Rajalakshmi et al. [71]	Smartphone retinal images graded with EyeArt software	301 patients	Any DR: 95.8% STDR: 99.1% RDR: 99.3%	Any DR: 80.2% STDR: 80.4% RDR: 68.8%	**PPV** Any DR: 89.7% STDR: 75.3% RDR: 74.6% **NPV** Any DR: 91.4% STDR: 99.3% RDR: 99.1%	–
Sarao et al. [72]	Images from a new confocal device versus fundus camera graded with AI software	144 patients (288 eyes)	Confocal device: 94.7% Fundus camera: 90.7%	Confocal device: 83.3% Fundus camera: 76.2%	–	–

*AI* artificial intelligence, *AUROC* area under the curve of the receiver operating characteristic, *DL* deep learning, *DR* diabetic retinopathy, *FDA* Food and Drug Administration, *IDP* Iowa Detection Program, *NPDR* non-proliferative diabetic retinopathy, *NPV* negative predictive value, *PPV* positive predictive value, *RDR* referable diabetic retinopathy, *STDR* sight-threatening diabetic retinopathy

^a^AUROC value lies between 0.5 (corresponding to a random guess) and 1.0 (indicating 100% sensitivity and specificity). ^b^Classified all screening episodes as “disease” or “ungradeable”

Fleming et al. (2010) affirmed that including automated algorithms to identify RDR lesions in digital fundus images is likely to be a cost-effective adjunct to manual grading. By adding algorithms for detection of exudates and blot hemorrhages to an MA detection algorithm, a significant increase in sensitivity of RDR detection was achieved, without there being an increase in the manual grading workload [58]. The automated software detected 100% of patients with proliferative or referable/observable background retinopathy and would reduce workload by 36.3% compared with entirely manual grading [59].

### Quality assurance

Service quality assurance reduces the probability of error and risk and helps professionals and organizations improve over time. Methods of quality assurance include post hoc evaluations of cost-effectiveness and accuracy of image grading [60]. In a South African study that included 261 ophthalmologists and optometrists, the Scottish Diabetic Retinopathy Grading Scheme was chosen because simplicity and clear cutoffs for referrals are vital for public sector eye clinics that have a high number of patients and limited resources [60]. The participants graded 90 retinal photographs as an external test of quality assurance, and the findings were: large disparities in grader performance; a general lack of specificity among screeners; and a mean diagnostic odds ratio of 12.3, which was considered to be at the low end of the range for a medium level of performance (10.13–22.24). The authors concluded that this test process was well accepted by participants, demonstrated safety of screening and highlighted areas in which more training is needed. The authors of this review were in agreement that local screening programs should follow national quality assurance standards to ensure a safe and effective service [60].

### Cost-effectiveness of screening

Cost-effective DR screening programs have been trialed, evaluated and fully implemented in many countries [9, 61]. In a retrospective review of data from the US Department of Veterans Affairs, Kirkizlar et al. (2013) determined that a telemedicine program for DR screening was cost-effective only for populations > 3500 and patients < 80 years of age [31]. In South Africa, researchers concluded that screening patients by mobile non-mydriatic digital fundoscopy in a primary care setting was cost-effective when images were captured by a trained technician and overseen by an ophthalmic nurse and the results were read by a medical officer [62]. In a public healthcare setting in Hong Kong, investigators determined that it was cost-effective to screen patients with diabetes for DR and age-related macular degeneration in the same session [63]. In a study of cost-effectiveness of screening and laser treatment for DR and macular edema in Malawi, Vetrini et al. (2018) determined that annual photographic screening with laser treatment for STDR and macular edema was cost-effective in terms of QALYs gained, given an 80% service utilization rate [64]. The program was most likely to be cost-effective when utilization was high and young patients (simulated age of 30 years) were screened [64].

Telemedicine screening systems have been cost-effective for monitoring patients with diabetes in rural and urban settings when compared with in-person screening or no screening. A retrospective chart analysis in Manitoba, Canada, which involved 4676 patients with diabetes, demonstrated average savings of Can$1007 (approximately US$800) per teleophthalmology examination [73]. Potential savings increased as more patients were examined [73].

### Screening intervals

There is some uncertainty about the optimal interval for DR screening. Tung et al. (2008) stated that annual screening for DR was medically and economically worthwhile in a Chinese population of patients with type 2 diabetes [74]. In England, combining two consecutive years of screening data revealed that annualized rates of progression to STDR were only 0.7% and 1.9% for those with no DR at either screening or with unilateral DR at the second screening, respectively. In contrast, the annualized rate was 11% in those with bilateral DR at both screenings. The authors noted that these estimates of the risk of future development of STDR could be used to inform decisions regarding screening frequency [75].

DR screening of a hypothetical cohort in rural southern India was shown to be cost-effective from a healthcare provider perspective at 2-year intervals, but not annually [76]. From a societal perspective, telescreening was cost-effective only as frequently as every 5 years [76]. Romero-Aroca et al. [77] suggested that screening for DR every 2.5 years is cost-effective, but the screening interval should be adjusted to the patient’s personal risk factors. Lund et al. [78] conducted individualized risk assessments with the aim of optimizing screening intervals. They showed that DR progression could be reliably predicted and that most patients have a < 5% risk of any DR progression in a given year. The authors noted that a screening interval of 20 months could be applied to these low-risk patients [78]. Similarly, a retrospective analysis of a screening program in Turin showed that less than 4% of 4294 patients with no DR at baseline progressed to RDR over 2 years, irrespective of other clinical variables, and none required immediate treatment by an ophthalmologist [79].

Scanlon et al. [80] determined that, in the absence of personalized risk stratification, it was most likely to be cost-effective to screen all patients every 3 years; annual screening of all patients was not cost-effective. In the context of a personalized screening paradigm for STDR, screening low-risk patients every 5 years, medium-risk patients every 3 years and high-risk patients every 2 years was the most cost-effective strategy. The authors noted that although the algorithm should be applicable in general, more work is needed to validate screening in various populations [80].

### Risk factors

DR risk has been found to be significantly associated with ethnicity, blood pressure, hemoglobin A1c (HbA1c) levels, duration of diabetes and pregnancy [35, 81]. Investigators in Portugal determined that the incidence of any DR and RDR and the DR progression rate were all related to duration of diabetes, age at diagnosis and use of insulin [82]. In a cross-sectional study of four urban US sites, DR was shown to be correlated with duration of diabetes, but not with smoking status, health insurance status or knowledge of HbA1c level [83]. Risk factors associated with DR in a large screening study in Spain included duration of diabetes, HbA1c levels, blood pressure and hypertension [84]. DR is also associated with the presence of other vascular complications that occur with diabetes, and the presence of such complications should be considered when evaluating a patient’s risk of the presence or progression of DR [85].

### Barriers to screening

In patients with diabetes, increased time from diagnosis to first screening episode correlates with more advanced retinopathy [86, 87]. In one study, RDR was detected in 2.3% of patients screened within 6 months and in 4.2% of patients screened 3 years or more after diagnosis [86]. Therefore, screening for DR should be done promptly after diagnosis and at regular risk-based intervals thereafter.

Numerous factors are known to affect attendance at DR screening [87–91]. In an established mobile retinal screening service in Scotland, poor attendance at screening was not linked to gender or to distance from the event, but was associated with younger age, longer duration of diabetes, smoking, social deprivation, and poor HbA1c and blood pressure control; the highest proportion of non-attenders were in urban areas [92]. Findings from seven UK screening programs demonstrated that patients aged 18–34 years were least likely to promptly attend screening after registration with the screening program, and these patients had a higher risk of RDR presence at initial screening [87]. In a study of screening uptake among 21,797 patients, uptake increased with each age stratum from 67% (age 12–39 years) to 88% (age 70–79 years) before declining to 79% in those aged ≥ 80 years [91]. Decline in the oldest patients may have reflected age-related issues such as limited access to screening services because of comorbidity or reduced mobility. Other factors known to lower uptake include poor awareness of the importance of screening, psychological factors (such as guilt due to poor diabetic control and fear of laser treatment) and practical barriers to attendance [91]. A UK study also confirmed that uptake rates were lower in more socioeconomically deprived groups; in contrast, practices in the most socioeconomically advantaged regions had the highest uptake [93]. Moreover, there was robust disparity in uptake among the practices, which may have been due to variability in the flexibility and availability of screening appointments offered [91].

In a US study of low-income patients and their healthcare providers and staffers, investigators found a striking lack of agreement in perceived barriers to screening [90]. Providers and staffers felt that transportation, language issues, cultural beliefs or myths, denial and fear were key barriers to DR screening, whereas patients indicated that financial burden and depression were the most common barriers [90]. Judah et al. [94] evaluated the effectiveness of financially incentivizing screening among patients in the NHS Diabetic Eye Screening Programme who had not attended an appointment for at least 2 years. There was no difference in attendance between the fixed incentive and control groups (relative risk, 0.70; 95% CI 0.35–1.39), and incentives reduced attendance compared with invitation letters in patients who were regular non-attenders. The authors emphasized that future work should focus on non-financial means of overcoming barriers to screening encountered by patients [94].

In a DR screening program at a publicly funded clinic in Alabama, only 30% of the 949 patients with diabetes adhered to interval recommendations for follow-up eye appointments [89]. The authors concluded that these programs are unlikely to meet public health goals unless adherence is promoted through adequate educational initiatives [89]. Similarly, findings of the Korean National Health and Nutrition Examination Survey indicated that only 36.3% of patients with diabetes had been screened in the previous year [95]. Patients in rural areas and those who were less educated (overall or about diabetes care) were found to be screened less often [95]. However, poor adherence has been reported in both rural and urban settings [2].

Geographic information system (GIS) mapping is useful for visualizing geographic access and barriers to eye care and may help identify underserved areas that would benefit from expansion of teleophthalmology screening programs [88]. In North Carolina, the authors used data on 1787 patients with diabetes who underwent retinal screening to develop qualitative GIS maps of patient and provider density around five telemedicine sites [88]. Results indicated that patient accessibility to healthcare professionals can be limited by geography and road networks. Primary care clinicians were somewhat uniformly distributed, but ophthalmologists were concentrated at urban centers. The authors emphasized that telemedicine has great potential for reducing barriers to care by connecting physicians with patients in rural, remote and underserved areas [88].

Another barrier to implementation of effective screening is delayed examination by a specialist. In a cross-sectional study in Ireland, 395 of 1542 patients were found to have some level of DR, and 11 had proliferative STDR [96]. Of these, 3 were given specialist appointments that were > 5 months from the original referral date [96].

### Screening for DME

Currently, there is a lack of evidence on screening specifically for DME; however, detection of DME is important because this condition is the leading cause of blindness in patients with type 2 diabetes [97]. One study from a DME screening program in Hong Kong demonstrated a high false positive rate (86.6%) and a low positive predictive value (13.4%) for screening of DME with mydriatic FP, if macular thickness was used to define the presence of macular edema [98]. This is the DME screening method currently applied in the UK and Hong Kong. The authors noted that it places large financial burdens on the healthcare system and can cause unnecessary psychological stress for patients because of the high false positive rate [98]. As noted earlier, performing OCT scans only on selected patients with suspected DME may reduce false positives and improve screening [22].

Dupas et al. [99] incorporated automated detection of DME alongside DR grading in 761 patients and noted sensitivity of 72.7% and specificity of 70.9% for DME risk. Prescott et al. [100] applied OCT alongside automated grading of fundus photographs to identify DME in 3170 patients who had positive results for DR in a prior screen involving conventional digital photography. This strategy yielded cost savings in both England and Scotland [100]. Olson et al. [101] considered the cost-effectiveness of adding OCT to a standard digital retinal photograph to detect macular edema and found that automated detection of patterns of photographic surrogate markers was superior to manual grading for detecting macular edema by OCT. The authors also determined that, by incorporating OCT into the screen prior to referral, costs could be lowered without any additional cases of macular edema being missed. They noted that worse visual acuity was associated with a fivefold higher prevalence of macular edema and therefore suggested including a functional analysis with the automated strategy [101].

## Summary: Fundamental principles of an effective DR screening program

The point of care, imaging methods, use of mydriasis, training of personnel and cost-effectiveness are important factors to consider when implementing DR screening programs. Standard retinal FP is used most frequently, but other methods such as UWF may be preferable, particularly if examinations are to occur without induction of mydriasis [17, 18]. Although no handheld device has demonstrated sensitivity and specificity comparable to seven-field examinations, such devices may be preferable from a telemedicine perspective and when barriers to standard screening exist. To reduce movement artifacts, both the handheld device and the patient’s head should be fixed [24–26].

Potential barriers to screening include age (uptake rates are lowest in children and younger adults, and in older adults with reduced mobility) [91], geography [95], financial constraints or socioeconomic status [90, 91, 93] and lower education level [95]. DR screening via telemedicine is effective [35, 36], can improve screening by reducing patient-perceived barriers to care [38, 39, 41], may improve follow-up [40] and appears to be cost-effective [37]. For any DR screening program to be effective, staff accreditation, appropriate IT infrastructure, continuing education and quality assurance procedures need to be in place and national standards should be followed [4, 60]. As part of training and quality assurance, a uniform system of grading should be in place, including a definition of RDR that is understood and identified by image graders [32, 52]. Although it is clear that using an estimate of future risk of DR, based on factors such as age and duration of diabetes, to determine screening intervals is warranted and likely to improve the cost-effectiveness of a screening program, methods for risk stratification still require standardization and validation [80].

The value of DR screening is now well established, but attempts to develop effective DME screening programs have, as yet, failed to achieve similar success [98]. However, including DME screening as part of DR evaluation may have tangible benefits [101].

Based on these considerations, the Vision Academy Working Group identified the following basic features for implementing an effective screening program for DR. We believe that these recommendations will be helpful to ophthalmology and diabetology communities.

- Examination methods must be suitable for the screening region, and DR classification/grading systems must be systematic and uniformly applied.
  - Although there is variability among studies in the number of image fields acquired (ranging from one to nine), two-field retinal imaging is sufficient for DR screening and is preferable to seven-field imaging.
  - There should be a definition of DR in place that is understood and used by screening staff to reliably identify RDR.
- In many countries/regions, screening can and should take place outside the ophthalmology clinic. Cost-effective telemedicine programs can be performed in a variety of alternative settings.
- Screening staff should be accredited and show evidence of ongoing training.
- Screening programs should adhere to relevant national quality assurance standards.
- Studies that use uniform definitions of risk to determine optimum risk-based screening intervals are required.
- Technology infrastructure should be in place to ensure that high-quality images can be stored securely to protect patient information.
- Although screening for DME in conjunction with DR evaluations may have merit, there is currently insufficient evidence to support implementation of programs solely for DME screening.
