# Correction to: Fundamental principles of an effective diabetic retinopathy screening program

**DOI:** 10.1007/s00592-020-01541-5

**Published:** 2020-05-18

**Authors:** Paolo Lanzetta, Valentina Sarao, Peter H. Scanlon, Jane Barratt, Massimo Porta, Francesco Bandello, Anat Loewenstein, on behalf of the Vision Academy, Bora Eldem, Alex Hunyor, Antonia Joussen, Adrian Koh, Jean-François Korobelnik, Paolo Lanzetta, Anat Loewenstein, Monica Lövestam-Adrian, Rafael Navarro, Annabelle A. Okada, Ian Pearce, Francisco J. Rodríguez, Giovanni Staurenghi, Sebastian Wolf, David T. Wong

**Affiliations:** 1 Department of Medicine – Ophthalmology, University of Udine, Piazzale S. Maria della Misericordia, 33100 Udine, Italy; 2 Istituto Europeo di Microchirurgia Oculare (IEMO), Udine, Italy; 3 Gloucestershire Hospitals NHS Foundation Trust, Gloucester, UK; 4 International Federation on Ageing, Toronto, Canada; 5 Department of Medical Sciences, University of Turin, Turin, Italy; 6 San Raffaele Scientific Institute, Milan, Italy; 7 Department of Ophthalmology Tel Aviv Medical Center, and Sackler Faculty of Medicine, Tel Aviv University, Tel Aviv, Israel

## Correction to: Acta Diabetologica 10.1007/s00592-020-01506-8

Authors would like to correct few errors in their publication which are listed below.

1. Study group name is corrected as “on behalf of the Vision Academy”.
2. In the section “Barriers to screening” reference {94} is removed.
3. Affiliation 1 is corrected as below
  Department of Medicine – Ophthalmology, University of Udine, Piazzale S. Maria della Misericordia, 33100 Udine, Italy
4. Correct version of Acknowledgements and Authors’ contributions is updated here.

### Acknowledgements

The Steering Committee of the Vision Academy who advised on the publication concept included the following members: Bora Eldem, Hacettepe University, Turkey; Alex Hunyor, University of Sydney, Australia; Antonia Joussen, Charité – Berlin University of Medicine, Germany; Adrian Koh, Eye & Retina Surgeons, Camden Medical Centre, Singapore; Jean-François Korobelnik, University Hospital of Bordeaux, France; Paolo Lanzetta, University of Udine, Italy; Anat Loewenstein, Tel Aviv Sourasky Medical Center, Israel; Monica Lövestam-Adrian, Lund University Hospital, Sweden; Rafael Navarro, Institute of Ocular Microsurgery, Spain; Annabelle A. Okada, Kyorin University School of Medicine, Japan; Ian Pearce, Royal Liverpool and Broadgreen University Hospitals NHS Trust, UK; Francisco J. Rodríguez, Fundación Oftalmológica Nacional, Colombia; Giovanni Staurenghi, University of Milan, Italy; Sebastian Wolf, University Hospital of Bern, Switzerland; and David T. Wong, St. Michael’s Hospital, University of Toronto, Canada. Bayer provided financial support in the form of funding for the Vision Academy and for medical writing to Porterhouse Medical Ltd. Bayer had no role in the design or conduct of this research. The views and opinions expressed are those of the authors and the Vision Academy, and not necessarily those of Bayer.

### Authors’ contributions

Study conception and design: Paolo Lanzetta. Acquisition of data: Paolo Lanzetta, Valentina Sarao, Peter H. Scanlon, Jane Barratt, Massimo Porta, Francesco Bandello, Anat Loewenstein. Analysis and interpretation of data: Paolo Lanzetta, Valentina Sarao, Peter H. Scanlon, Jane Barratt, Massimo Porta, Francesco Bandello, Anat Loewenstein. Drafting of manuscript: Paolo Lanzetta, Valentina Sarao, Peter H. Scanlon, Jane Barratt, Massimo Porta, Francesco Bandello, Anat Loewenstein. Critical revision: Paolo Lanzetta, Valentina Sarao, Peter H. Scanlon, Jane Barratt, Massimo Porta, Francesco Bandello, Anat Loewenstein.

5. Ref. [65] corrected version updated here

Abràmoff MD, Niemeijer M, Suttorp-Schulten MSA, Viergever MA, Russell SR, Van Ginneken B (2008) Evaluation of a system for automatic detection of diabetic retinopathy from color fundus photographs in a large population of patients with diabetes. Diabetes Care 31(2):193–198

The original article has been corrected.

